# Bilateral Large Orbital Lymphoma With Proptosis

**DOI:** 10.7759/cureus.36548

**Published:** 2023-03-22

**Authors:** Dharshini Balasubaramaniam, Sujaya Singh, Fazilawati A Qamarruddin, Kavitha Saravanamuthu

**Affiliations:** 1 Ophthalmology, Universiti Malaya, Kuala Lumpur, MYS; 2 Ophthalmology, Hospital Tengku Ampuan Rahimah, Klang, MYS

**Keywords:** chemosis, proptosis, ocular adnexal lymphoma, diffuse large b-cell lymphoma, orbital lymphoma

## Abstract

Diffuse large B-cell lymphoma (DLBCL) is the most common lymphoid malignancy in adults. It is an aggressive malignancy and requires a multidisciplinary approach with various modalities which include chemotherapy, radiotherapy as well as immunotherapy. A 63-year-old Malay male patient with underlying type 2 diabetes mellitus, hypertension, ischemic heart disease, and stage II chronic kidney disease presented with a one-month history of bilateral eye proptosis associated with lid swelling and red eye. He also complained of progressive right eye blurring of vision. Visual acuity was counting fingers on the right and 6/18 on the left. On examination, the relative afferent pupillary defect was negative. There was bilateral eye proptosis, conjunctival chemosis, and restricted extra-ocular movement in all gazes. There was also exposure keratopathy over the right eye, and intraocular pressure was raised. Bilateral cervical and axillary lymph nodes were palpable. A computerized tomography scan of the brain and orbit revealed bilateral orbital masses with no bony erosions. An incisional biopsy over the upper lid confirmed the diagnosis of diffuse large B-cell lymphoma with multiple myeloma-1 (MUM-1) positivity which defines the activated B-cell subtype (ABC). He was co-managed with a hematologist and was commenced on the rituximab-cyclophosphamide, doxorubicin, vincristine, prednisone (R-CHOP) chemotherapy regime. Bilateral eye proptosis, chemosis, and restriction of extra-ocular movement resolved after the completion of treatment. However, right eye vision remains poor as the patient developed central self-sealed corneal perforation with iris plugging which has healed with scarring. Diffuse large B-cell orbital lymphoma is a fast-growing and aggressive tumor, hence early diagnosis and prompt multi-disciplinary treatment are crucial for a good outcome.

## Introduction

Diffuse large B-cell lymphoma (DLBCL) is the most common non-Hodgkin lymphoma. Although it commonly involves the lymph nodes, extranodal DLBCL presents in 40% of individuals with common sites of involvement being the gastrointestinal tract, skin, and soft tissue [1]. Diffuse large B-cell lymphoma of the ocular adnexal region is relatively rare, accounting for only 8% to 13% of cases, and includes the orbit, eyelids, conjunctiva, lacrimal gland, and lacrimal sac [2]. Out of these, the orbit and conjunctiva appear to be the most commonly affected site [1].

Diffuse large B-cell lymphoma shows a higher incidence in older patients, with 70% of patients being older than 50 years of age and only approximately 10% being younger than 40 years. The median age of presentation is the 7th decade [1,3]. Munch-Petersen et al. reported a slight female predominance, however, Li et al. and Stacy et al. reported a male predominance [1,3,4].

In this report, we describe a rare case of bilateral large orbital DLBCL presenting with proptosis. 

## Case presentation

A 63-year-old Malay gentleman with underlying type 2 diabetes mellitus, hypertension, ischemic heart disease and stage II chronic kidney disease presented with a one-month history of bilateral eyelid swelling associated with proptosis. He also complained of progressive right eye blurring of vision and red eye for a one-week duration. He denied fever, night sweats, or loss of weight. There was also no history of trauma.

**Figure 1:**
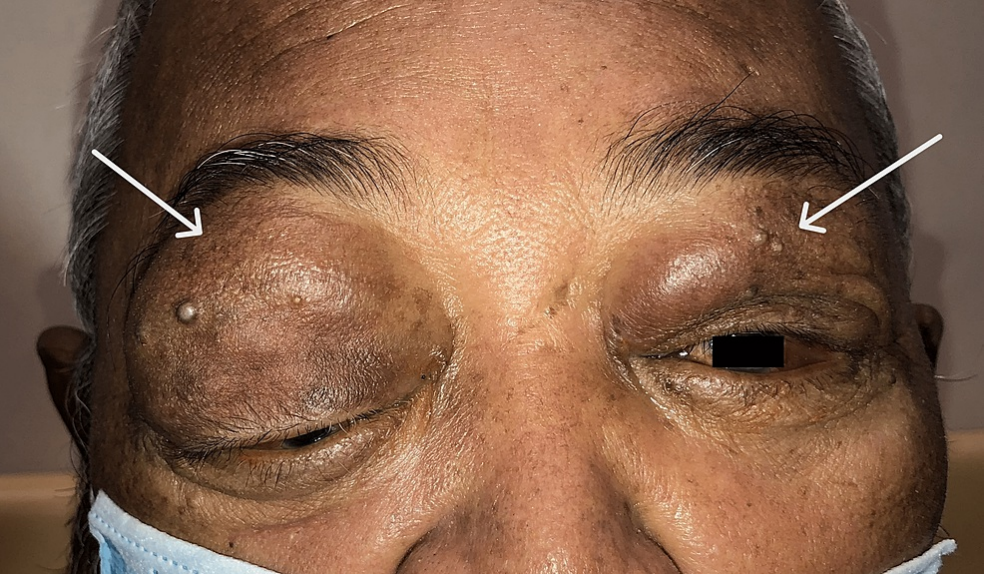
Bilateral upper lid swelling and proptosis

**Figure 2:**
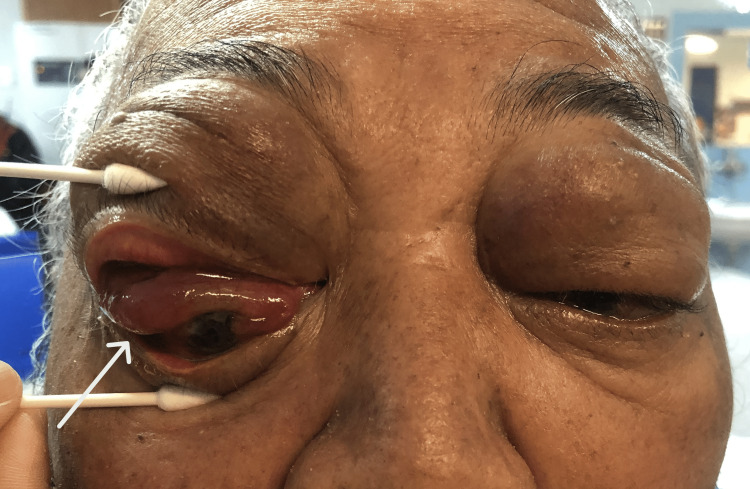
Right-eye chemosis with "salmon patch" appearance of conjunctiva

On systemic examination, the Glasgow coma scale was 15/15. However, the patient was slightly lethargic. Vital signs were stable. Cervical and axillary lymph nodes were palpable. Ocular examination revealed a visual acuity of counting with fingers over the right eye and 6/18 over the left eye. The relative afferent pupillary defect was negative and pupils were 3mm, round, and reactive bilaterally. Bilateral eyes were proptosed, measuring 22mm on the Hertel exophthalmometerover the right eye, and 18mm over the left eye. There were bilateral upper lid multiple lobulated firm masses with mechanical ptosis and right-sided inferior dystopia (Figure 1). Right-eye conjunctiva was chemosed with a "salmon patch" appearance of the upper conjunctiva (Figure 2). There was a central epithelial defect with exposure keratopathy over the inferior half of the cornea and the anterior chamber was deep and quiet. There was no rubeosis. Intraocular pressure was 18mmHg. 

The left eye was mildly chemosed. The cornea was clear with punctate epithelial erosions and the anterior chamber was deep and quiet. There was no rubeosis. Intraocular pressure was 22mmHg. Both eye fundus examinations revealed a pink optic disc with a well-defined margin and a cup disc ratio of 0.7. Retina was flat with a normal macula and no choroidal folds. Vessels were slightly tortuous. Extraocular movements were restricted in all directions of gaze.

**Figure 3:**
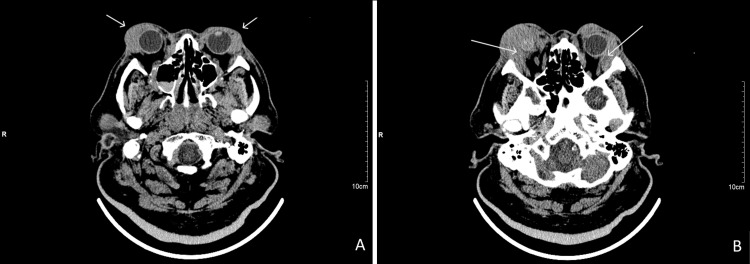
Non-contrasted axial-cut CT brain and orbit revealed bilateral extra-conal (A) and intra-conal (B) masses arising from the lacrimal gland with involvement of the extraocular muscles (A and B)

An urgent computerized tomography scan of the brain and orbit revealed bilateral intra-conal and extra-conal masses arising from the lacrimal gland with the involvement of the extraocular muscles. There was no evidence of optic nerve compression or bony erosions (Figure 3).

**Figure 4:**
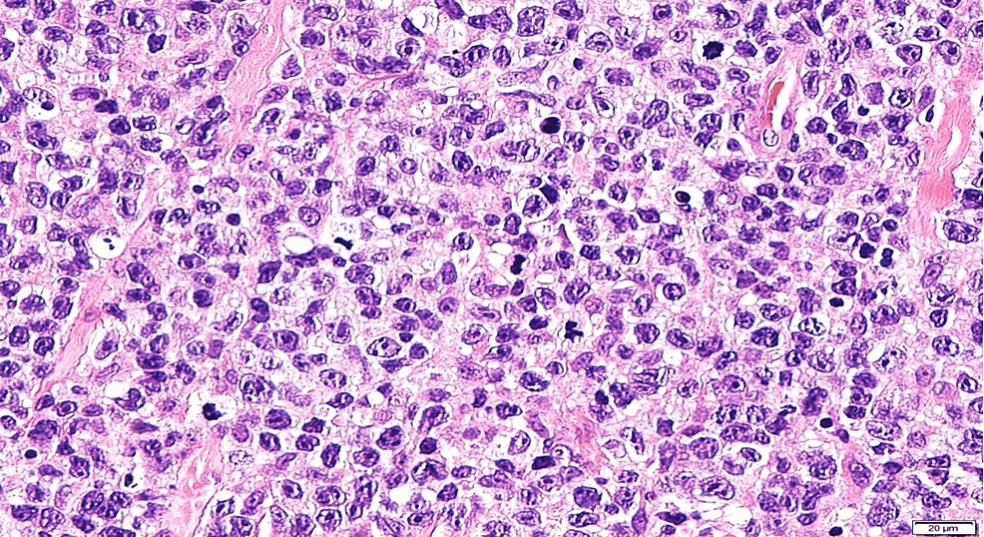
Histopathological slide from the right upper eyelid tissue using hematoxylin and eosin stain Histopathology reveals tissue diffusely infiltrated by atypical lymphoid cells arranged in sheets. Observed are cells that are medium to large in size, irregular nuclear membranes, coarse chromatin, and prominent nucleoli with scanty cytoplasm. Mitotic figures and apoptotic bodies are numerous.

An incisional biopsy done over the bilateral upper lid confirmed the presence of DLBCL with multiple myeloma-1 (MUM-1) positivity which defines the activated B-cell (ABC) subtype (Figure 4).

The patient was diagnosed with DLBCL-stage IV E, bulky disease. The patient was co-managed with a hematologist and was commenced on rituximab-cyclophosphamide, doxorubicin, vincristine, prednisone (R-CHOP) chemotherapy regime. He responded well to the first cycle of chemotherapy. However, during his hospital stay the patient was diagnosed to be Covid-19 positive. In view of his multiple co-morbidities, critical systemic condition, and low blood pressure, he opted for palliative chemotherapy.

**Figure 5:**
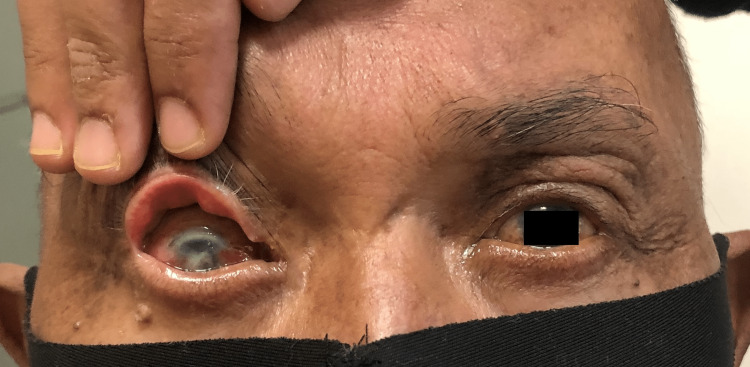
Resolved bilateral proptosis, chemosis, and upper lid swelling after two cycles of R-CHOP R-CHOP: Rituximab-cyclophosphamide, doxorubicin, vincristine, prednisone

The patient completed his palliative chemotherapy and is now stable. Bilateral eye proptosis, lid swelling, and chemosis have resolved (Figure 5). However, right eye vision remains poor as the patient developed central self-sealed corneal perforation with iris plugging which has healed with scarring.

## Discussion

Orbital DLBCL is a rare but aggressive malignancy. Patients usually present with a rapidly growing swelling involving one or more lymph nodes or extranodal sites, ocular irritation, epiphora, exophthalmos, diplopia, ptosis, dry eye, and decreased visual acuity. About one-third of patients present with B symptoms which are fever, weight loss, and night sweats or symptoms related to organ(s) involvement [2,3].

The average symptom duration is approximately 9.6 months [1]. Interestingly, our patient presented with a one-month history of progressively increasing upper lid swelling, which showed a rather rapidly growing tumor. According to Munch-Petersen et al., the most common clinical sign of ocular DLBCL is an ocular mass (82.0%) followed by globe displacement (39.0%) which are both seen in our patient [1]. Other signs include chemosis, edema, restricted ocular movement, diplopia, and ptosis [1,2]. Patients commonly present with unilateral disease unlike in our case where the patient presented with bilateral eye involvement [1,2].

Diffuse large B-cell orbital lymphoma is a locally invasive tumor that affects adjacent orbital structures such as periorbital bones, sinuses, and the intracranial region. Involvement of the eyeball and optic nerve is very rare [2]. The diagnosis of DLBCL requires histopathological examination of a tissue biopsy and is tested for several receptors such as cluster of differentiation (CD)79a, CD20, MUM-1, and BCL-2, for which our patient tested positive. However, prior to biopsy and testing, a complete ophthalmological examination followed by a systemic examination is needed. Imaging studies such as CT or MRI brain and orbit are performed to identify the size and location of the tumor along with the involvement of the adjacent structures. An excisional biopsy is mandatory to confirm the diagnosis and subtype classification [2,3]. For staging purposes, full-body positron emission tomography-computed tomography (PET-CT), MRI, and a bone marrow biopsy can be performed.

The World Health Organization classifies DLBCL according to cell-of-origin i.e., germinal centre-B(GCB)-cell-like and ABC-like subtypes. However, 10% to 15% of cases remain unclassifiable [4]. This involves the examination of morphology and testing of antibodies directed against CD3, CD5, CD20, and CD79a in order to differentiate between B- and T-cell lymphomas. Once B-cell lymphoma is confirmed, tests are run to detect antibodies against BCL2, BCL6, CD10, CD23, CD30, Cyclin D-1, MUM-1, and κ and λ light chains [2]. Subtype classification is important to decide treatment options and prognosticate outcomes. Patients with the GCB subtype generally have a better prognosis than the ABC subtype [3,5]. This explains the rapidity and deterioration of the condition in our patient, which finally required palliative treatment.

Two staging systems are commonly used to stage the disease: the Ann Arbor staging system and the tumor, node, metastases (TNM) staging system by The American Joint Committee on Cancer (AJCC). The Ann Arbor system classifies the disease based on the number of sites of involvement and their relation to the diaphragm, the presence of B symptoms, and the presence of extranodal disease [3]. On the other hand, the TNM staging system is staged based on the size and extent of the primary tumor (T), the presence of local lymph nodes (N), and the evidence of metastasis (M).

Managing DLBCL requires a multi-disciplinary team that involves the ophthalmologist, hematologist, oncologist, and radiotherapist. A few crucial factors to be considered are the subtype of the disease, the extension of the tumor, the presence of metastasis, prognostic factors, and the extent of ocular involvement [1].

Treatment options include chemotherapy, external radiotherapy, surgery, steroids, monoclonal antibodies, and stem cell transplant. Chemotherapy is the most common treatment of choice in the management of high-grade or disseminated orbital lymphoma. The frequently practiced regime is CHOP. However, other combination regimes are hyper-cyclophosphamide, vincristine, doxorubicin, dexamethasone (CVAD), methotrexate, cytarabine; and cyclophosphamide, vincristine, and prednisone (CVP) [1,2].

Immunotherapy using monoclonal antibodies is also commonly advocated as a combination with chemotherapy [1,2]. Rituximab (R) is a human/mouse anti-CD20 antibody that binds the CD20 antigen on normal cells and tumor cells causing it to lyse. The introduction of the R-CHOP regime has significantly improved the survival rates of lymphoma patients.

Steroids are also given in combination with chemotherapy. Prednisone, which is the common steroid of choice, is known to have a direct anti-cancerous effect on acute lymphatic leukemia and lymphoma. However, the exact mechanism is unknown.

Besides this, radiotherapy can be used as a primary treatment option to eradicate the tumor, to reduce tumor size prior to surgery, or in combination with chemotherapy and immunotherapy [2]. Commonly reported side effects with high doses of radiotherapy more than 30Gy to 35Gy include cutaneous reactions, cataracts, dry eye, macular degeneration, retinopathy, and corneal ulceration secondary to xerophthalmia. Low-dose radiotherapy that is 4Gy to 8Gy shows a good local response with minimal side effects [1,2].

Surgery is not commonly preferred for the treatment of DLBCL with only 30% of cases being treated with total or partial excision of the tumor [2]. It is opted for only in cases of massive or bulky tumors where a reduction in tumor size is warranted. Following surgery, chemotherapy or radiotherapy is used as an adjunct treatment.

## Conclusions

Diffuse large B-cell orbital lymphoma is a fast-growing and aggressive tumor. Hence, early diagnosis and prompt multi-disciplinary treatment are imperative for a good outcome. Besides tissue diagnosis, subtype classification is also important in deciding treatment options and prognosticating outcomes. The current standard therapy for patients with DLBCL is the R-CHOP regimen in which the majority of patients are cured. However, a small number respond poorly to standard therapy. In our case, the patient presented early, but due to the aggressive nature of the disease and multiple comorbidities, critical systemic condition, and low blood pressure, his treatment ended up being palliative.
